# Routine Antenatal Echocardiography in High-Prevalence Areas of Rheumatic Heart Disease: A WHO-Guideline Systematic Review

**DOI:** 10.5334/gh.1318

**Published:** 2024-04-25

**Authors:** Samuel Seitler, Mahmood Ahmad, Sanjali Anil Chu Ahuja, Malik Takreem Ahmed, Alexander Stevenson, Tamar Rachel Schreiber, Prem Singh Sodhi, Hiruna Kojitha Diyasena, Osarumwense Ogbeide, Sankavi Arularooran, Farhad Shokraneh, Miryan Cassandra, Eloi Marijon, David S. Celermajer, Mohammed Y. Khanji, Rui Providencia

**Affiliations:** 1 Royal Free Hampstead NHS Trust, Royal Free London NHS Foundation Trust, Pond St, London NW3 2QG, UK; 2 Barts and The London School of Medicine and Dentistry Turner St, London E1 2AD, UK; 3 GKT School of Medical Education, King’s College London, UK; 4 Department of Cardiology, High Wycombe Hospital, Queen Alexandra Rd, High Wycombe HP11 2TT, UK; 5 GENEs health and social care evidence SYnthesiS unit, Institute of Health Informatics, University College London, UK; 6 Evidence Synthesis, Systematic Review Consultants LTD, Nottingham, UK; 7 Hospital Ayres de Menezes, São Tomé, ST; 8 Paris Cardiovascular Research Centre, INSERM U970, European Georges Pompidou Hospital, Paris, FR; 9 Department of Cardiology, European Georges Pompidou Hospital, Paris, FR; 10 The University of Sydney, Sydney, AU; 11 Department of Cardiology, Royal Prince Alfred Hospital, Sydney, AU; 12 Cardiology Department, Barts Heart Centre, Barts Health NHS Trust, London, UK; 13 Newham University Hospital, Barts Health NHS Trust, Glen Road, Plaistow, London E13 8SL, UK; 14 NIHR Barts Biomedical Research Centre, William Harvey Research Institute, Queen Mary University, London EC1A 7BE, UK

**Keywords:** ultrasound, pregnancy, rheumatic heart disease, rheumatic fever, cardiac disease

## Abstract

**Background:**

Rheumatic Heart Disease (RHD) is the most common cause of valvular heart disease worldwide. Undiagnosed or untreated RHD can complicate pregnancy and lead to poor maternal and fetal outcomes and is a significant factor in non-obstetric morbidity. Echocardiography has an emerging role in screening for RHD. We aimed to critically analyse the evidence on the use of echocardiography for screening pregnant women for RHD in high-prevalence areas.

**Methods:**

We searched MEDLINE and Embase to identify the relevant reports. Two independent reviewers assessed the reports against the eligibility criteria in a double-blind process.

**Results:**

The searches (date: 4 April 2023) identified 432 records for screening. Ten non-controlled observational studies were identified, five using portable or handheld echocardiography, comprising data from 23,166 women. Prevalence of RHD varied across the studies, ranging from 0.4 to 6.6% (I^2^, heterogeneity >90%). Other cardiac abnormalities (e.g., congenital heart disease and left ventricular systolic dysfunction) were also detected <1% to 2% of cases. Certainty of evidence was very low.

**Conclusion:**

Echocardiography as part of antenatal care in high-prevalence areas may detect RHD or other cardiac abnormalities in asymptomatic pregnant women, potentially reducing the rates of disease progression and adverse labor-associated outcomes. However, this evidence is affected by the low certainty of evidence, and lack of studies comparing echocardiography versus standard antenatal care.

**Prospective Registration:**

PROSPERO 2022 July 4; CRD42022344081 Available from: https://www.crd.york.ac.uk/prospero/display_record.php?RecordID=344081.

**Research question:**

‘In areas with a high prevalence of rheumatic heart disease, should handheld echocardiography be added to routine antenatal care?’

## Introduction

Rheumatic heart disease (RHD) is the most common cause of valvular heart disease worldwide, impacting millions, especially in low- and middle-income countries [1]. RHD is a long-term consequence of untreated and recurrent acute rheumatic fever (ARF), an autoimmune response to infection with Group A Streptococcus (Strep A), with ARF-associated carditis leading to lasting damage to heart valves which can eventually lead to heart failure and death [2].

Pregnancy results in major changes in the cardiovascular system including increases in blood volume, heart rate, and stroke volume, such that cardiac output increases up to 50% when compared to non-pregnant levels [3]. These changes can often unmask previously unrecognized cardiac disease or exacerbate clinical symptoms in women with known disease. It has been reported that pulmonary edema occurs in approximately 60% of women with significant mitral stenosis when the vascular volume is near its peak, at 30 weeks [4].

Data from 5,739 pregnancies on the Registry of Pregnancy and Cardiac disease (ROPAC), which included data from 138 centres in 53 countries (low-, mid-, and high-income) over a 12-year period showed that congenital heart disease and valvular heart disease were the two most prevalent diagnoses, accounting for 57% and 29% of the total, respectively. RHD accounted for 56% of cases of valvular heart disease [5]. A higher proportion of RHD was observed in two single tertiary-centre studies from South Africa, a high-prevalence country, with RHD present in up to 80% of pregnant women with heart disease [6,7].

Undiagnosed or untreated RHD can complicate pregnancy and is a significant factor for non-obstetric morbidity and an important cause of maternal death [8]. Preterm birth has been reported in 23% of cases, intrauterine growth restriction in 21%, low birthweight (below the 10th percentile) in 28%, and unfavorable fetal outcome (i.e., spontaneous or therapeutic abortion, or stillbirth) observed in 11% of pregnancies from a tertiary centre high-risk obstetrics/cardiology clinic [8]. Involvement of the mitral valve has an important prognostic impact, with a subgroup analysis of women with mitral valve disease in the ROPAC registry (mitral stenosis in two thirds and isolated mitral regurgitation in the remaining) revealing that heart failure occurred in one fourth of all mitral valve disease women [9]. This rate increased to one third and nearly one half, in cases of moderate and severe mitral stenosis, respectively. An intervention during pregnancy was required in 4.1%, either percutaneous balloon mitral commissurotomy (n = 14) or mitral valve replacement (n = 2). Complications, mainly pulmonary oedema, and occurring in the third trimester, were observed in >50% in a series of 138 consecutive pregnant women with mitral stenosis [10], and 14.5% (n = 20) required balloon valvulotomy. Four women (1.0%) died within the first six months in the ROPAC registry [9], and seven (3.3%) died in a South African cohort [6].

The heightened risks faced by pregnant women with RHD demand greater emphasis on timely detection and prompt management to prevent complications to mother and child. Early diagnosis is of importance to prevent disease progression, with a systematic review of nine randomized and quasi-randomized studies showing reduction of ARF recurrence with secondary prevention with penicillin [11]. A recent trial including 818 cases of subclinical mild RHD showed that secondary antibiotic prophylaxis reduced the risk of progression at two years [12].

Two-dimensional (2D) echocardiography is established as the mainstay imaging modality for the diagnosis and monitoring of RHD [13] and ARF [14]. Echocardiography can be performed using handheld, portable, or stationary devices. Handheld echocardiography has advantages for screening, due to simplicity and being lightweight, but does not possess the advanced high-tech imaging features of heavier portable and stationary devices. Handheld 2D echocardiography has an emerging role in screening for RHD [15]. Mirabel and colleagues have shown that focused echocardiogram can be performed with reliable accuracy and reproducibility following relatively simple training of non-experts (i.e., two nurses) [16]. This approach is followed by a confirmatory echocardiographic study if the screening results are suggestive of RHD. Advantages and issues with each of the different echocardiography options for screening RHD are discussed in detail in Appendix 1 (Supplementary Material).

Clinical diagnosis of ARF and RHD may miss a significant percentage of cases, with auscultation only detecting murmurs in 6 out of 27 cases with subclinical RHD in Zühlke et al. [17], and missing RHD and ARF in 16% and 24%, respectively, in 281 children with febrile illness but not presenting with clinical criteria for ARF [18]. The role of echocardiography for screening pregnant women in high RHD prevalence areas is still uncertain. This systematic review will critically analyse the available evidence on the use of transthoracic echocardiography (handheld, portable, and non-portable) for screening pregnant women for RHD in high-prevalence areas.

## Methods

This systematic review was performed to address one of the questions (Question 11) of the World Health Organization Update of Guidelines on Prevention and Management of ARF and RHD: ‘In areas with a high prevalence of RHD: should handheld echocardiography be added to routine antenatal care?’

### Original protocol

The detailed protocol was pre-published on PROSPERO – 2022/CRD42022344081 [19], and available as supplementary material (Annex 1). We did not identify any studies comparing routine antenatal screening with echocardiography versus standard antenatal care. Consequently, we were not able to assess the diagnostic test accuracy of echocardiography vs standard antenatal care for detecting RHD in pregnancy in high prevalence areas or assess the impact of this approach on pregnancy-related outcomes.

### Modified protocol

After discussion with the Guideline Committee in March 2023, it was accepted that the inclusion criteria and study design would have to be broadened so observational studies using handheld echocardiography, or any other form of echocardiography (i.e., standard echocardiography), in the absence of a control group would also be considered eligible. This change aimed to gain further knowledge on the rate of important cardiac findings obtained through echocardiographic screening in pregnancy in high-prevalence populations.

The population of interest for this review was: pregnant women in areas with high prevalence of RHD; we used the data from Watkins et al. 2017 [20] to define high-prevalence areas. The Index Test was echocardiography during routine antenatal care. No comparator or reference test was required. The outcomes of interest were:

- Carditis in ARF, based on revised Jones Criteria by American Heart Association [13], or other criteria locally in use at the time of the study
- RHD, based on the 2012 World Heart Federation (WHF) criteria for echocardiographic diagnosis [14], or other criteria locally in use at the time of the study
- Adverse events (deaths, obstetrics complications, other)
- Time to diagnosis
- Acceptability to provider and patient

Studies were eligible for the purpose of this systematic review when describing findings of echocardiographic screening in pregnant women in areas of high prevalence of RHD. No age restrictions were applied. Studies not reporting on echocardiographic findings were not considered eligible.

Searches were run, documented, and reported by a senior information specialist (FS) on Embase via Ovid SP (1974–present) and MEDLINE via Ovid SP (1946–present). The original search strategy for diagnostic test accuracy studies is described in detail in Appendix 1 and 2 (Supplementary Material). This was subsequently revised and rerun on 4 April 2023, using the search terms ‘Pregnan*’ and ‘Echocardiogr*’ and ‘Rheumatic’, also targeting non-controlled, prevalence studies.

The search results were imported into EndNote 20. After removal of duplicates, the remaining records were then imported into Rayyan, for double-blind screening by two reviewers (SS & SACA). The blinding was inactivated when the screening was finished to resolve the conflicts by a third reviewer (MA).

The following data were extracted from all studies (MA) and double-checked by an independent reviewer (RP).

- Study characteristics: authors, year of publication, country, study design, sample size, study period, setting, patient selection (random/consecutive), follow-up period.
- Patient characteristics: patient type, age, sex, highest level of education, presence of cardiovascular risk factors (hypertension, diabetes mellitus), HIV status, known cardiac disease, gestational age, gestation number, primigravida, presence of symptoms, or New York Heart Association functional status.
- Index test details: Handheld echography device used (type – handheld, portable, stationary device; model), level of experience of the sonographer, screening protocol, and diagnostic criteria.
- Outcomes: Carditis-ARF, RHD, any adverse event (deaths, complication), time to diagnosis, and acceptability to provider and patient
- Other echocardiographic findings, n and%: left ventricular (LV) systolic dysfunction (reported as LV ejection fraction value, class, or other utilized measure), LV hypertrophy, right ventricular dilation, and presence and severity of mitral regurgitation, mitral stenosis, aortic regurgitation, aortic stenosis, and pulmonary hypertension. Data on detection and type of congenital heart disease were also extracted.

We contacted the authors of the studies on an as-required basis to obtain the data or information.

Quality assessment of studies was done by two researchers (SS & MA) using the Newcastle Ottawa scale, which comprises three domains: selection, comparability, and outcome/exposure [21]. Studies were classified as low, moderate, or high quality according to the following criteria: studies scoring a total of 7 to 8 were considered low risk of bias; studies with a score of 6 were considered to have a medium risk of bias; studies scoring 5 points or less were considered to have a high risk of bias. With respect to selection, studies were considered to have a low, medium, or high risk of bias if they scored 3, 1 to 2, or 0 points, respectively [22]. With respect to comparability, studies were considered to have a low, medium, or high risk of bias if they scored 2, 1 or 0 point, respectively. Finally, with respect to outcome, studies scoring 3, 2, or 1 point, were, respectively, considered to have a low, medium, or high risk of bias. Disagreements between the two researchers were resolved by consensus or via a third party (RP).

Statistical heterogeneity was quantified using the I^2^ statistic, which describes the percentage of total variation across studies due to heterogeneity rather than chance. Values of <25%, 25% to 50%, and >50% are by convention classified as low, moderate, and high degrees of heterogeneity, respectively.

We summarised % rates with 95% confidence intervals across studies in a forest plot. Meta-analysis using a random-effects model was planned if heterogeneity as per I^2^ was not considered high (i.e., if I^2^ < 50%) [23]. Funnel plots were used for evaluating the presence of publication bias and traced for outcomes of interest including ≥10 studies [24].

## Results

**Figure 1:**
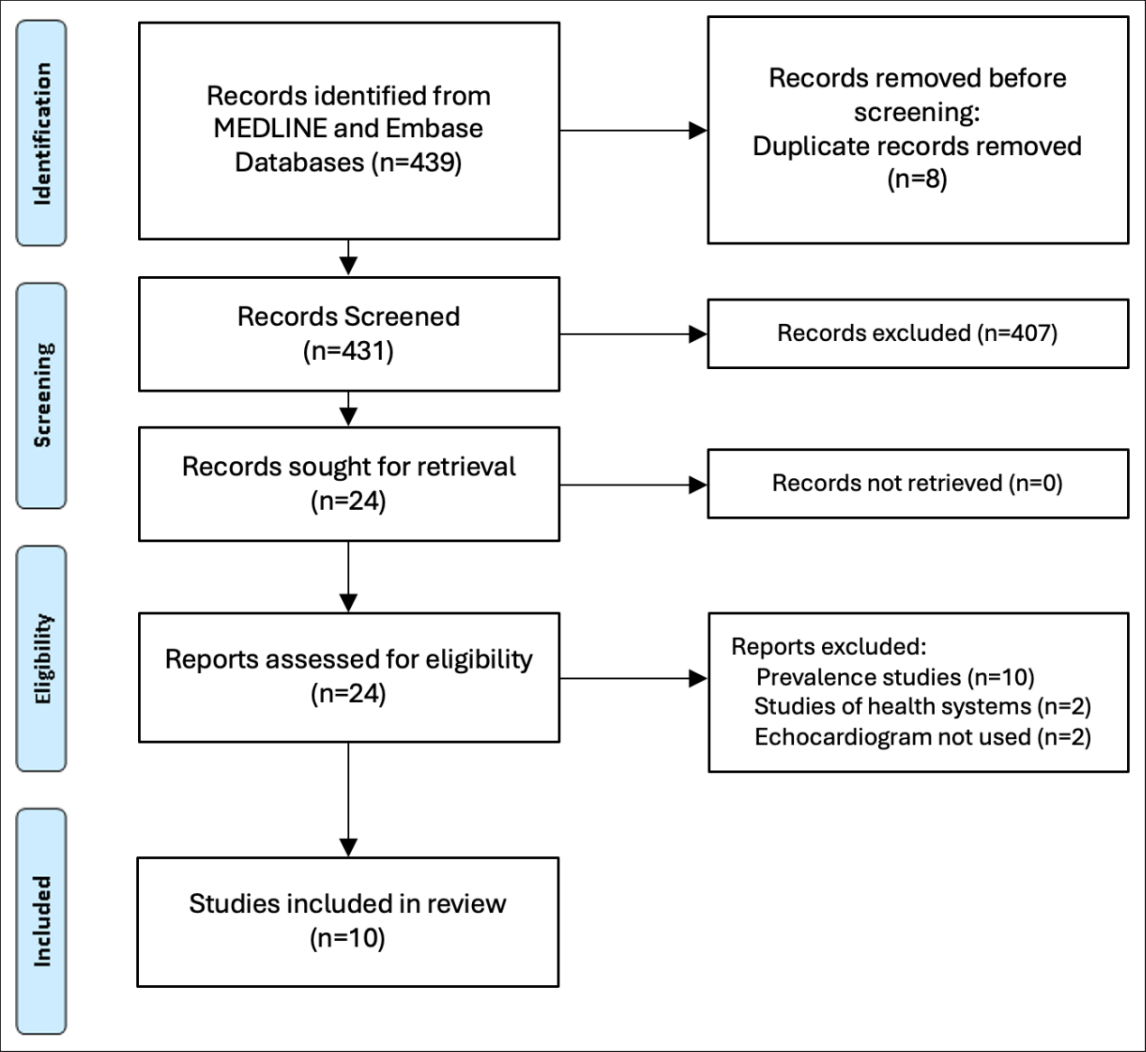
PRISMA flow diagram.

The searches identified 4,565 records, but we were unable to find any controlled studies looking at handheld echocardiography versus standard transthoracic echo or standard clinical care to diagnose RHD in pregnant women (Figure S-1; Supplementary Material). Following the revision of the inclusion criteria in the abovementioned WHO Guideline Committee meeting (March 2023), a new search on 4 of April 2023 yielded additional 432 records for screening. Ten non-controlled observational studies were identified using echocardiography for screening of cardiac disease in pregnant women from high prevalence areas of RHD [25,26,27,28,29,30,31,32,33,34], five using portable or handheld echocardiography [25,26,27,28,29] (Figure 1). One study [30] was published as abstract, and the remaining as full text. Four studies were conducted in sub-Saharan Africa [25,26,28,31], four studies were conducted in India [29,30,32,34], one in Brazil [27] and one in Turkey [33]. The sample sizes were highly variable, ranging from 300 [32] to 14,275 [34], with a combined total of 23,166 women across studies.

In one study all approached women accepted to participate [25]. In Nascimento et al. nearly 50% of women with positive findings on the screening echo failed to attend the recommended follow-up echocardiogram despite the multiple contact attempts [27]. In Snelgrove et al., of 810 potentially eligible women, only 601 consented and accepted to undergo echocardiography [28]. No other studies provided information on acceptability to patent of provider.

Demographic information for the included women is shown in Table 1. Maternal age was consistent throughout the studies, as were pre-existing medical co-morbidities. Maternal HIV status was recorded in two of the studies. Mean gestational age was similar in four of the studies, with most women being scanned in the second or third trimester. Four studies were single-centre studies conducted in a tertiary maternity care setting [28,30,33,34,35]. The remainder were multi-centre studies, coordinated across several clinics in a single city [26] or several states [27,29]. Two studies had no participants with known cardiac disease at baseline [25,33], two studies had less than 1% [26,27], and two [29,30] had up to 5% of pregnant women with known disease at baseline. Bozkaya et al. only recruited women in the first trimester [33]. In three studies most women had an echocardiogram between the 22nd and 25th week of gestation [26,27,28]. In Otto et al. most echocardiograms were performed at 30 weeks or later [25]. Alshaqri et al. was the only study to also include post-partum women in the analysis [29].

**Table 1:** Demographic and obstetric data.


STUDY ID	COUNTRY	DESIGN	SETTING	SAMPLE SIZE	AGE (YEARS) (MEAN ± SD)	HIGHEST LEVEL OF EDUCATION	HYPERTENSION, n (%)	DIABETES, n (%)	HIV n (%)	KNOWN CARDIAC DISEASE n (%)	GESTATIONAL AGE AT FIRST VISIT, WEEKS (MEAN SD)	PRIMIGRAVIDA, n (%)

**Studies using Handheld or Portable Echocardiography**												

**Otto 2011**	Eritrea	Prospective cross sectional	4 clinics in single city	348	26.9 (±6)	NA	0	NA	NA	0	30.9 ± 6.7	NA

**Beaton 2019**	Uganda	24-month prospective longitudinal investigation	2 health centres, 1 regional referral hospital	3506	24.09 (N/A)	University Degree: 87 (2.5%)	6 (1.7%)	NA	141 (4.0%)	2 (0.06%)	24 (median)	359 (10.2%)

**Nascimento 2021**	Brazil	13-month prospective longitudinal investigation	22 primary care centres	1112	27 ± 8 years	NA	50 (4.5%)	27 (2.4%)	NA	9 (0.8%)	22 ± 9	NA

**Snelgrove 2021**	Kenya	Cross sectional study	Antenatal clinic at tertiary maternity centre	601	26.6 years (SD 5.7)	University degree: 82 (13.6%)	1 (0.2%)	1 (0.2%)	45 (7.5%)	27 (4.5%)	25.3 ± 9.5	NA

**AlSharqi 2022**	India	cross sectional study	10 hospitals across three states-India	301	25.4 (18–42)	University degree: 31 (10.6%)	NA	NA	NA	17 (5.6%)	NA	183 (60.8%)

**Screening using Standard Echocardiography or unspecified echocardiographer**												

**Selvarani 2014**	India	6 months Cross sectional study	Antenatal clinic at a tertiary maternity centre	1125	23.41	NA	NA	NA	NA	NA	NA.	55.73%

**Bacha 2019**	Ethopia	Cross Sectional Study	Antenatal clinic at tertiary maternity centre	398	27.0 (±4.6)	College/University 49 (12.31%)	8 (2%)	7 (1.8%)	NA	NA	NA	NA

**Gomathi 2019**	India	4-month cross-sectional study	Cardiology department at tertiary center	300	NA	NA	NA	NA	NA	0	NA	186 (62%)

**Bozkaya 2020**	Turkey	12-month cross-sectional study	Tertiary delivery center	900	27.4 (±5.8)	NA	0	0	0	0	NA/first trimester	NA

**Patel 2021**	India	36 month long cross sectional study	Antenatal care at two large hospitals	14275	NA	NA	NA	NA	NA	NA	NA	NA


Echocardiography was conducted as part of antenatal care in all studies. The images were acquired by operators and interpreted with a variety of experience. Otto et al. [25] and Beaton et al. [26] used specially trained medical students and nurses, respectively, while Snelgrove et al. [28] used an accredited sonographer. The acquired images were then locally reviewed by cardiologists or trained sonographers [25,26,28]. Alsharqi et al. [29] employed trained obstetricians and Nascimento et al. [27] used healthcare workers to acquire the images. These two studies exported DICOMs internationally for remote analysis by experts. Echocardiography was performed by a cardiologist in Bozkaya et al. [33].

Scanning protocols differed among the studies, with Nascimento et al. adapting a simplified seven- view echocardiographic protocol for handheld devices, and Alsharqi et al. using the MaatHRI focused image acquisition protocol (Table 2). The criteria used for diagnosis of RHD were not uniform. Four studies [26,28,31,33] used the 2012 WHF criteria for the echocardiographic diagnosis of RHD [14] (Table 3). Otto et al. [25] conducted their study prior to the development of these criteria but used similar morphological abnormalities for diagnosing RHD. Two studies [27,29] used criteria from the American Society of Echocardiography. The remaining studies did not define the echocardiographic criteria for RHD diagnosis.

**Table 2:** Echo protocols and findings.


	PROTOCOL	OPERATORS	LVSD, n (%)	LV HYPERTROPHY, n (%)	PULMONARY HYPERTENSION, n (%)	RV DILATATION, n (%)	SYMPTOMS/NYHA FUNCTIONAL CLASS AT ENTRY	CONGENITAL HEART DISEASE (ANY) n

**Studies using Portable or Handheld Echocardiography**								

**Otto 2011**	Full TTE with Doppler; Vivid-I GE Healthcare	Trained Medical students	Moderate LVSD: 1 (0.29%)	0	0	0	Asymptomatic	Any: 1 (0.3%) ASD

**Beaton 2019**	Focussed Echo; Vivid-Q GE Healthcare or Philips ClearVue 350	Nurses	DCM, Mild LVSD: 1 (0.03%)	0	Any: 1 (0.03%)	0	Cases with echo findings: I–7 (13.4%) II–41 (78.8%) III–4 (7.7%) IV–0 (0%)	Any: 1 (0.03%) Large secundum ASD: 1

**Nascimento 2021**	Focussed 7-view protocol; Vscan GE Healthcare and Vivid-Q & IQ GE Healthcare, if positive scan	Healthcare workers	Mild/moderate LVSD: 1 (0.1%)	2 (0.2%)	0	3 (0.3%)	37.7% (419) had dyspnoea	0

**Snelgrove 2021**	Focussed Echo; Vivid-Q GE Healthcare	Cardiac sonographer	0	0	Any: 1 (0)	0	10.3% (62) had unspecific symptoms	Any: 2 (0.3%) Unroofed CS: 1; PDA: 1

**AlSharqi 2022**	Focussed protocol; Lumify Philips Healthcare	Obstetricians	LVEF 45–54% 8.4% (25) LVEF 30–44% 8.8% (26) LVEF <30% 4.7% (14)	0	0	19/285 (6.7%)	Cases with echo findings (n = 172): I–13 (7.8%) II–37 (22.2%) III–19 (11.4%) IV–98 (58.6%)	0

**Screening using Standard Echocardiography or unspecified echocardiographer**								

**Selvarani 2014**	Full TTE with Doppler; Echocardiographer not specified	NA	Any: 1 (0.1%) DCM: 1	0	0	0	All asymptomatic “except for a few in NYHA class II”	Any: 24 (2.1%) ASD: 7; VSD: 2; MVP: 3; Bicuspid Ao: 3; Corrected: 9

**Bacha 2019**	Full TTE with Doppler; Vivid-E9 GE Healthcare	NA	Any: 1 (0.3%) Peri-natal CM: 1	5 (1.3%)	Any: 15 (3.8%) Mild: 11 Moderate: 2 Severe: 2	NA	NA	0

**Gomathi 2019**	Full TTE with Doppler; Echocardiographer not specified	NA	NA	NA	NA	NA	Asymptomatic	Any: 7 (2.3%) ASD: 2; MVP: 3; Pulm Stenosis: 1; Aortic Coarct: 1

**Bozkaya 2020**	Full TTE with Doppler; – Vivid S5 System, GE Healthcare	Cardiologist	0	NA	NA	0	Asymptomatic	Any: 9 (1.0%) ASD: 8; PDA (1)

**Patel 2021**	“Screening echocardiogram”; Echocardiographer not specified	NA	Cardiomyopathy: 66 (0.5%) DCM: 30 (0.2%) LVEF < 55% 36 (0.3)	27(0.18%)	NA	NA	NA	Any: 63 (0.4%) ASD or PFO: 43; VSD: 4; PDA (7), Bicuspid Aortic valve (9)


Legend: LVSD – left ventricular systolic dysfunction; ASD – atrial septal defect; CS – coronary sinus; PDA – patent ductus arteriosus; CM – cardiomyopathy, MVP – mitral valve prolapse; Pulm – pulmonary; Coartct – coarctation. Other findings: Alsharqi et al. reported 4 thrombi; Bozkaya et al. also reported significant cases of non-rheumatic valve disease: moderate pulmonary stenosis (1), moderate AR (1), moderate MR (5), and severe MR (1).

**Table 3:** Rheumatic heart disease findings on screening echo.


	CRITERIA USED	RHD ANY n, %	MITRAL REGURGITATION, n (%)	MITRAL STENOSIS (ANY), n (%)	AORTIC REGURGITATION (ANY), n (%)	AORTIC STENOSIS (ANY), n (%)

**Studies using Portable or Handlehd Echocardiography**						

**Otto 2011**	WHO consensus statement 2001	Any: 16 (4.6%) Definite: 8 Nondefinite: 8	Any: 12 (3.6%) Mild: 7 Mid/Moderate: 2 Moderate: 2 Moderate/Severe: 1	0	Any: 6 (1.7%) Mild: 6	0

**Beaton 2019**	WHF	Any: 51 (1.5%) Mild: 31 Moderate: 14 Severe: 6	Any: 45 (1.3%) Mild: 28 Moderate: 15 Severe: 2	Any: 3 (0.1%) Mild: 2 Moderate: 1	Any: 7 (0.2%) Mild: 5 Moderate: 1 Severe: 1	0

**Nascimento 2021**	Adapted ASE criteria for major heart disease	Any: 12 (1.1%)*	Any: 12 (1.1%) Mild to moderate: 11 Moderate: 1	Any: 1 (0.1%) Mild: 1	Any: 3 (0.3%) Mild: 3	Any: 1 (0.1%) Mild: 1

**Snelgrove 2021**	WHF	Any: 3 (0.5%)	Any: 1 (0.2%) Moderate: 1	Any: 2 (0.33%) Severe: 2	Any: 1 (0.2%) Moderate: 1	0

**AlSharqi 2022**	ASE/EACVI recommendations	Any: 20 (6.6%)	‘mitral valve involvement’: 20 (6.6%)	Significant: 17 (5.6%)	‘aortic valve involvement’ 3 (1%)	Significant: 1 (0.3%)

**Screening using standard echocardiography or nonspecified echocardiographer**						

**Selvarani 2014**	NA	Any: 17 (1.5%)	Any: 1 (0.1%)	Any: 14 (1.2%)	Any: 3 (0.03%)	NA

**Bacha 2019**	WHF	Significant: 9 (2.3%)	Any: 4 (1%) Moderate to Severe: 2 NS: 2	Moderate to Severe: 3 (0.8%)	Moderate to Severe: 2 (0.5%)	Moderate to Severe: 2(0.5%)

**Gomathi 2019**	NA	Any: 12 (4.0%)	Any: 4 (1.3%)	Any: 4 (1.3%) Moderate: 3 Not specified: 1	Any: 1 (0.3%) Not specified: 1	Any: 1 (0.3%) Not specified: 1

**Bozkaya 2020**	WHF	Any: 26 (2.9%)	Any 24 (2.7%) Mild: 20 Moderate: 4	Mild: 2 (0.2%)	0	0

**Patel 2021**	NA	61 (0.4%)	Any: not specified; ‘mitral valve involvement’: 61	Number not specified, but ‘90% were mild to moderate, and 67% had Wilkins score <8’	NA	NA


*RHD on screening was suspected in 36 women (3.2%), but only 56 of the 100 women who screened positive had a confirmatory echocardiogram.

### Rheumatic heart disease

Due to high heterogeneity (I^2^ > 90%) across the observational studies, data were not pooled, and a narrative description is presented below.

**Figure 2:**
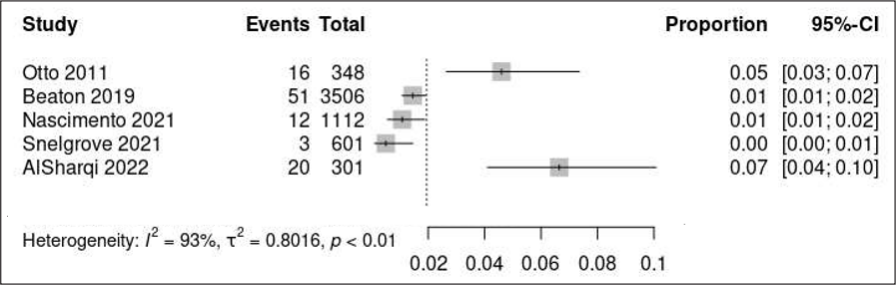
Prevalence of RHD in studies using portable or handheld echocardiography.

**Figure 3:**
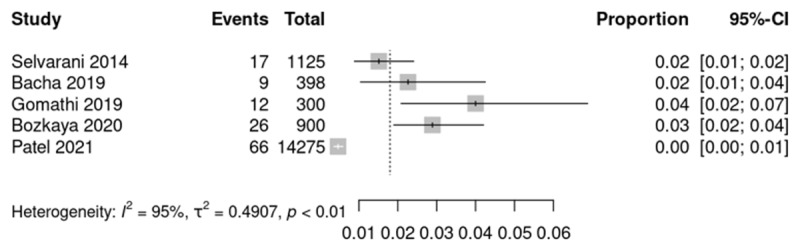
Prevalence of RHD in studies using stationary or non-specified echocardiography.

Studies from different global regions were identified, with RHD prevalence ranging from 0.5% in Kenya [28], 1.1% in Brazil [27], 1.5% in Uganda [26], 2.3% in Ethiopia [31], 2.9% in Turkey [33], 4.6% in Eritrea [25] to 6.6% in India [29]. Prevalence of RHD in studies conducted in India varied: Patel et al 0.4% [34], Selvarani et al 1.5% [30], Gomathi et al 2.6% [32] (Figures 2 and 3, and Tables 2 and 3).

Otto et al. classified their results into three groups, based on pre-defined criteria that predated the 2012 WHF classification (supplementary material – Table S-1) into: definite RHD (n = 8, 2.3%); non-definite RHD (n = 8, 2.3%); and no structural abnormalities. Across the definite and non-definite groups, the mitral valve was the most frequently affected (in 12 of 16 cases). All participants in the study were asymptomatic and none reported known previous RF [25].

Nascimento et al. demonstrated the benefits of portable echo as a screening tool for RHD. Suspected RHD was observed in 36 (3.2%) of 1,112 pregnant women scanned with handheld devices, utilizing a simplified echo protocol [27], and confirmed using more sophisticated portable machines in a smaller subset of women, all with mitral valve involvement and two with additional aortic valve compromise. Prevalence of positive screening findings was comparable in the first (8.9%), second (9.7%) and third (7.2%) trimester [27].

Beaton et al. identified heart disease in 58 women, corresponding to a community prevalence of 1.5% (95% CI 1.3% to 2.1%), with 51 out of 58 cases (87.9%) attributed to RHD [26]. Alsharqi et al., using handheld echocardiography, reported RHD in 20 women (6.6%), all of whom had mitral valve involvement, but no further information was provided regarding the severity of the valvulopathy [29]. Snelgrove et al. identified only 3 cases of RHD (0.5%) [27].

Severe RHD was a rare finding across all studies. Beaton et al. identified 31 women (60.8%) with mild, 14 (24.1%) with moderate, and 6 (11.8%) with severe valvular involvement. Nearly 97% of cases (i.e., all but two) of RHD were new diagnoses. This was the only study providing prospective follow-up of the women diagnosed with RHD during screening, with cardiovascular complications occurred in 51.8% (95% CI 39.0 to 64.3) of these women (heart failure in one third, pulmonary hypertension in one tenth and 5% with arrhythmia), and cardiovascular medication was required in over half [26]. One quarter of the identified women with heart disease were considered either high risk or moderate risk by the combined cardiology/obstetric team. Caesarean delivery was recommended for one woman at the national referral centre, delivery at the regional centre was recommended for seven and six additional women were referred for delivering in hospital [26]. Snelgrove et al. found that none of the three women identified with RHD had a prior formal diagnosis or knowledge of existing CVD disease, despite mitral stenosis being classified as severe in two of the cases [28]. Bozkaya et al. identified mild-to-moderate valve disease in 92.3% of the RHD cases [33]. Patel et al. described the highest number of women with RHD but did not stratify the distribution in any way [34].

### Other findings

Among studies using handheld echocardiography, left ventricular systolic dysfunction (LVSD) was an uncommon finding in most of the studies. Two studies reported no cases of LVSD [28,33] and three reported less than 0.5% prevalence of mild or moderate LVSD [25,26,27]. The exception was Alsharqi et al., who identified 51 (16.9%) cases of mild/moderate LVSD and 16 (4.7%) cases of severe LVSD from the 301 women imaged [29]. These results were skewed by the inclusion criteria, which comprised a large cohort of women with suspected heart failure (over 50% were NYHA IV at recruitment).

Several studies identified undiagnosed congenital heart diseases in their participants, most commonly intracardiac shunts. Otto et al. and Beaton et al. identified one case each of atrial septal defect [25,26]. Gomathi et al. identified two cases of ASD [32]. Snelgrove et al. detected one case of patent ductus arteriosus, and one case of unroofed coronary sinus [28]. Prevalence in other studies ranged from 1.0% (33), to 2.1% (30) or 2.3% (32) (details in Table 3).

No studies reported on detecting carditis in pregnant participants with clinical presentation compatible with ARF, or time to diagnosis.

### Quality assessment

Study quality varied across studies, with those using portable or handheld echocardiography scoring higher: four out of five studies were judged as low risk and one study [25] was considered medium risk (Table 4). The remaining studies were of lower quality: two were considered low [33] and medium risk [31], all remaining papers were considered high risk [30,32,34].

**Table 4:** Newcastle Ottawa scale for quality assessment of cross-sectional studies.


STUDY ID	SELECTION			COMPARABILITY	OUTCOME		TOTAL	RESULT
		
	REPRESENTATIVENESS OF THE SAMPLE	SAMPLE SIZE	NON- RESPONDENTS	Ψ	ASSESSMENT OF OUTCOME	STATISTICAL TEST		

**Studies using Portable or Handheld Echocardiography**								

**Otto 2011**	*	-	*	**	**	-	6	Medium Risk

**Beaton 2019**	*	*	-	**	**	*	7	Low Risk

**Nascimento 2021**	*	*	-	**	**	*	7	Low Risk

**Snelgrove 2021**	*	*	-	**	**	*	8	Low Risk

**AlSharqi 2022**	*	-	*	**	**	*	7	Low Risk

**Screening using Standard Echocardiography or nonspecified echocardiographer**								

**Selvarani 2014**	-	*	-	*	**	-	4	High Risk

**Bacha 2019**	*	*	-	**	**	-	6	Medium Risk

**Gomathi 2019**	-	-	-	-	**	-	2	High Risk

**Bozkaya 2020**	*	*	-	**	**	*	7	Low Risk

**Patel 2021**	*	*	-	-	**	-	4	High Risk


Ψ Comparability of subjects across studies with enough information provided on study design, analysis, and confounding factors (**); information provided on 2 of the 3 previously mentioned factors (*).

Assessing the three Newcastle-Ottawa scale domains in isolation, all studies but one [32], which was considered high risk, were classified as medium risk for selection bias. Regarding comparability, seven studies were considered low risk [25,26,27,28,29,31,33], and 3 were considered high risk [30,32,34]. Finally, with respect to outcome, five studies were considered low risk [26,27,28,29,33], and the remaining were medium risk.

**Figure 4:**
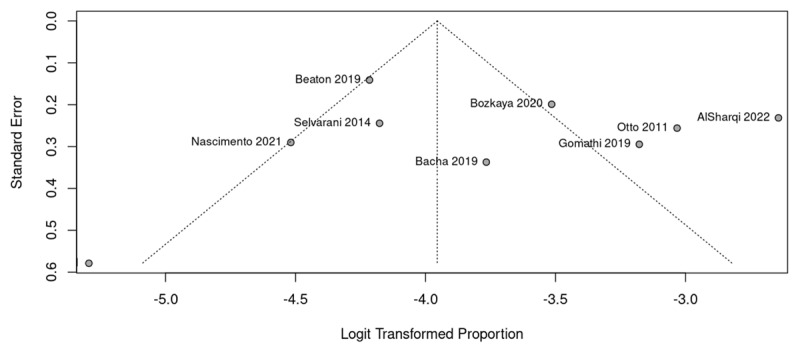
Funnel-plots for assessment of publication bias.

Certainty of evidence was considered very low. All studies were observational, and certainty was downgraded on the basis of concerns with risk of bias, inconsistency, imprecision, and publication bias (Figure 4). Justification for the decisions is provided in Table 5.

**Table 5:** Assessment of Certainty of Evidence using the GRADE approach.


NO OF STUDIES	RISK OF BIAS	INCONSISTENCY	INDIRECTNESS	IMPRECISION	PUBLICATION BIAS	CERTAINTY

10	Serious	Serious	Low risk	Serious	Serious	⨁◯◯◯Very low

	5 of 10 studies had medium to high risk of bias as per the Newcastle Ottawa scale	Inconsistency was very high: I^2^ = 95%	Study population of pregnant women in high prevalence countries in all studies	The detection rate of RHD was <1% in 2 studies	Asymmetric funnel plot, with Four studies are outside the 95%CI limits	


## Discussion

Our review shows that screening the antenatal population using handheld or portable echocardiography can identify an important proportion of women with previously undiagnosed RHD living in high-prevalence areas. The observed prevalence varied across the included studies and different global regions. This is not unexpected and may be due to wide variation of RHD prevalence in some geographical areas, as well as the different criteria for diagnosing and reporting RHD across studies. These findings are in-keeping with recent data estimating that the prevalence in countries where RHD remains endemic is >1%, and greatest in women of childbearing ages [7,34].

A large proportion of the RHD detected was mild in severity, which is generally well tolerated in pregnancy [9]. Although RHD was the most common finding, other clinically relevant conditions, such as congenital heart disease were detected. Some women had LVSD, pulmonary hypertension and high-risk left-sided valve lesions (e.g., aortic valve area <1.5 cm^2^, mitral valve area <2 cm^2^, or moderate to severe mitral regurgitation), which are included as part of widely accepted risk stratification schemes for women and cardiovascular disease such as CARPREG-II (Table S-2) [35], and the modified WHO classification of maternal cardiovascular risk (Table S-3) [36]. This is of relevance has it shows that besides being important for identifying women in need for secondary antibiotic prophylaxis, echocardiography as part of antenatal screening may be of importance for early detection and planning of pregnancy care in a significant number of women with undetected high-risk cardiovascular conditions living in high-prevalence areas.

The WHO has defined 13 recommendations for maternal and fetal assessment as part of their recommendations for antenatal care (Table S-4 – Supplementary Material) [37]. These include testing for HIV in high prevalence areas, screening for tuberculosis in areas with a prevalence > 1/1000 and an early ultrasound, before 24 weeks of gestation. According to the latest UNAIDS report, prevalence of HIV among adults aged 16 to 45 in Eastern and Southern Africa, and Western and central Africa, was 5.9 and 1.1%, respectively [38]. The early ultrasound may detect fetal anomalies in 1.2% [39], and multiple pregnancies in 1.7% of gestations [40]. These rates are comparable to the prevalence figures we described for RHD screening in our review.

Portable and handheld echocardiography screening may provide a more accessible strategy in these low-income settings where standard stationary transthoracic echocardiography is not easily accessible. There were variations in the strategy used for implementing echocardiography screening the studies we identified. Three studies trained nurses and/or obstetricians for obtaining an echocardiogram, and having the images interpreted remotely by trained cardiologists [26,27,29]. This strategy seems feasible and therefore might be easier to implement where antenatal care is largely led by community-based clinics, such as in Brazil [27]. Obstetricians, alongside nurses and midwives, perform WHO’s recommended antenatal ultrasound in high-prevalence areas for RHD [41] and may constitute potential alternatives upon receival of appropriate training in regions where cardiologists are sparse or lacking.

Timing for echocardiographic screening in pregnancy is still a matter of debate, and further research is needed to address this matter. Earlier screening has the potential advantage of improved acoustic window and adjusting pregnancy management planning in case changes are detected. Data from Beaton et al. provides evidence that antenatal screening with portable echocardiography at median of 24 weeks gestation can impact upon antenatal care and delivery planning [26]. However, we did not identify any studies comparing routine echocardiographic antenatal screening versus standard antenatal care that could provide direct evidence on the impact such an intervention might have upon maternal, fetal, and neonatal outcomes. This is an important knowledge gap that requiring further investigation.

Dealing with screening findings implies additional resource availability and utilization, as follow-up and need for further assessment and imaging of incidental findings may be required. Also, the findings may cause anxiety for the patient and relatives. Cost-effectiveness data were absent in the included studies. False positives and false negatives may be important points to factor, as these can potentially add to health system’s costs, but may be less of a problem with echocardiography than with cardiac auscultation.

By targeting pregnant women with suspected heart failure, Alsharqi et al. detected the highest rate of RHD, 6.6% [29], suggesting that, in regions where lack of resources could be a barrier for global screening, screening of higher risk groups with higher chances of findings could constitute an alternative. However, the best way to detect high-risk women remains to be clarified as studies in our review detected an important rate of RHD, ranging from 0.4 to 4.0% [25,32], in asymptomatic women. Not only could some of these women be high-risk and become symptomatic only later in pregnancy, as reported in the ROPAC registry [9], but also, missing these cases could deny them the chance of being offered secondary antibiotic prophylaxis to prevent progression of disease [12]. Studies focusing on patient selection vs global screening, applied to the local reality of the area where screening programs are being considered, are of importance to address this uncertainty.

The quality of most studies was low overall, and that was the main limitation of this systematic review. Most of the studies included in our review did not report the severity or type of valvular involvement. Data from the ROPAC registry shows that severe mitral stenosis is an independent risk factor for adverse fetal outcomes [9]. Interestingly, even in the ROPAC registry, severity of mitral of mitral stenosis was not classified in 20% of participants. Future studies and registries should provide more detailed information on degree of valvular involvement, and on RHD stage as per the 2023 World Heart Federation echocardiographic diagnosis guidelines [42]. Several factors could have contributed to the heterogeneity in the observed prevalence of RHD across studies: differences in the selected populations (e.g., some studies included only asymptomatic women or women without history of diagnosed cardiac disorders, whilst others included patients with suspected heart failure, or even with surgically corrected heart disease), utilized echocardiographer model, utilized criteria (WHF 2012 or other), and level of reporting (e.g., some studies only reported the more advanced cases of RHD). Most importantly, operator experience, which is key for an operator-dependent procedure like echocardiography, and background (cardiologists, obstetricians, sonographers, or other health professionals) varied across studies. Only two studies utilized handheld echocardiography, but with accessibility and ease of use, evidence for this technology is likely to grow.

## Conclusion

The observational studies identified in our review show that using echocardiography as part of routine antenatal care may detect between 0.4 to 6.6% of undiagnosed of RHD among pregnant women in high-prevalence areas. Reasons for the observed differences across studies need to be further clarified. Only two studies utilized handheld echocardiography in this setting, highlighting the need for more research in the field.

Evidence in this review is affected by the very low certainty of evidence, heterogeneity, lack of controlled trials (of handheld or portable echocardiography vs. standard stationary echocardiography), or cost-effectiveness studies.

## Additional File

The additional file for this article can be found as follows:

10.5334/gh.1318.s1 Supplementary Material. Figure S1, Tables S1 to S4 and Appendices 1 to 3.
