# Supplementary material for: Routine Antenatal Echocardiography in High-Prevalence Areas of Rheumatic Heart Disease: A WHO-Guideline Systematic Review
Source: Glob Heart. 2024 Apr 25;19(1):39. doi: 10.5334/gh.1318 (PMC11049603; doi:10.5334/gh.1318)
Supplement: Supplementary Material. — Figure S1, Tables S1 to S4 and Appendices 1 to 3. [file gh-19-1-1318-s1.pdf]

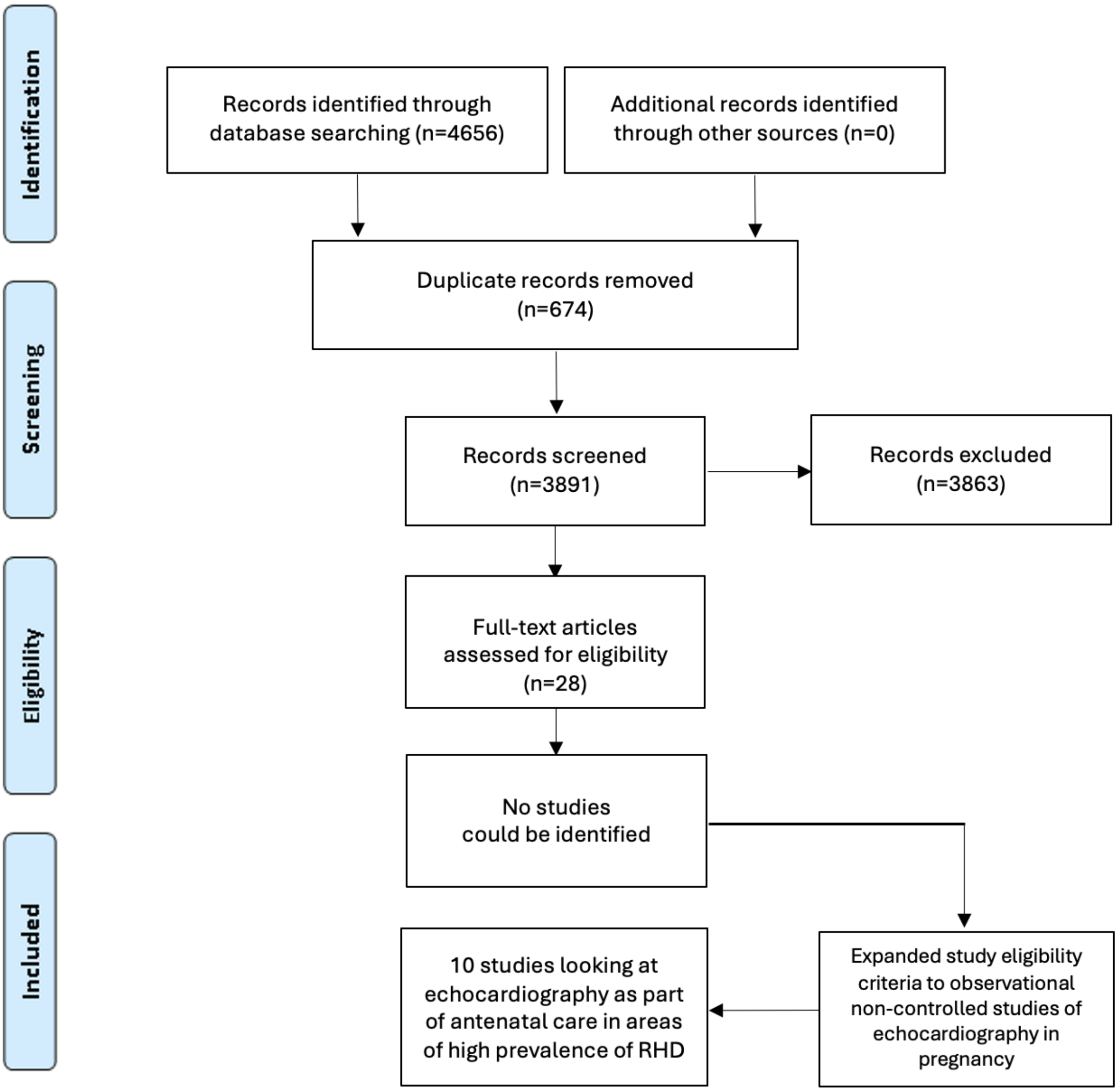

Figure S-1. PRISMA flow diagram illustrating the screening process.

Table S-1. Definition of Definite and non-Definite RHD by Otto et al. 2011

Definite RHD	-Thickening of the mitral valve leaflets or chordae together with a leak of any degree<br>-Obvious leaflet restriction even in the absence of any leak<br>-Mitral stenosis<br>-Aortic regurgitation of any degree with thickening of one or more leaflets<br>-Stenosis of a tricuspid aortic valve<br>-Tricuspid and pulmonary valve regurgitations of at least degree 2/4 with additional valvular thickening<br>-Grade 3–4 tricuspid and pulmonary regurgitations in absence of any other causes
Non-Definite RHD	Mitral and aortic valves which appeared somewhat thickened, but where a final assessment was difficult even in the presence of a regurgitation.

Table S-2. CARPREG-II risk score predictors

	**Predictors**
Patient history	Cardiac events or arrhythmias prior to pregnancy<br>Baseline NYHA functional class III/IV<br>No cardiac interventions prior to pregnancy
Physical exam	Cyanosis (saturations <90% at rest)
Specific lesions	Mechanical valvs<br>Coronary artery disease<br>High risk aortopathy
Imaging	Systemic ventricular dysfunction<br>High-risk left-sided valve lesion or left ventricular outflow tract obstruction<br>Pulmonary hypertension
Delivery of care	Late first antenatal visit

Note: The authors of the CARPREG-II risk score suggested other variables to consider when estimating pregnancy risk: rare or understudied cardiac conditions, other maternal comorbidities (i.e., advanced maternal age, hypertension, obesity), medications (i.e., anticoagulants), other cardiac test results (cardiopulmonary testing or magnetic resonance imaging), fertility therapy, patient compliance and patient access to care and quality of care.

Table S-3. Modified World Health Organization (mWHO) classification of risk of pregnancy in cardiovascular disease

mWHO class	Pregnancy risk & required action	Cardiac conditions
I	**Risk no higher than general population**	• Uncomplicated, small, or mild<br>- pulmonary stenosis<br>- ventricular septal defect<br>- patent ductus arteriosus<br>- mitral valve prolapse with no more than trivial mitral regurgitation<br>• Successfully repaired simple lesions, e.g.:<br>- ostium secundum atrial septal defect<br>- ventricular septal defect<br>- patent ductus arteriosus<br>- total anomalous pulmonary venous drainage<br>• Isolated ventricular extrasystoles and atrial ectopic beats
II	**Small increased risk of maternal mortality and morbidity**	• II if otherwise well and uncomplicated:<br>- unoperated atrial septal defect<br>- repaired tetralogy of Fallot<br>- most arrhythmias<br>• II or III depending on individual<br>- mild left ventricular impairment<br>- hypertrophic cardiomyopathy<br>- native or tissue valvular heart disease not considered WHO I or IV<br>- Marfan syndrome without aortic dilatation<br>- heart transplantation
III	**Significant increased risk of maternal mortality and morbidity**<br><br>**Expert cardiac and obstetric pre-pregnancy, antenatal and postnatal care required**	- mechanical valve<br>- systemic right ventricle (e.g., congenitally corrected transposition, simple transposition post Mustard or Senning repair)<br>- post-Fontan operation<br>- cyanotic heart disease<br>- other complex congenital heart disease
IV	**Pregnancy contraindicated: very high risk of maternal mortality or severe morbidity**<br><br>**Termination should be discussed**<br>**If pregnancy continues, care as for class 3**	• Pulmonary arterial hypertension of any cause<br>• Severe systemic ventricular dysfunction<br>- NYHA III–IV or LVEF <30%<br>• Previous peripartum cardiomyopathy with any residual impairment of left ventricular function<br>• Severe left heart obstruction<br>• Marfan syndrome with aorta dilated >40 mm

Table – S4. Recommended interventions for routine antenatal care by the World Health Organization: recommendations for maternal and fetal assessment

Recommendation	Type
- Full blood count testing is the recommended method for diagnosing anaemia in pregnancy	Context-specific
- Midstream urine culture is the recommended method for diagnosing asymptomatic bacteriuria in pregnancy	Context-specific
- Clinical enquiry about the possibility of intimate partner violence should be strongly considered	Context-specific
- Hyperglycaemia first detected at any time during pregnancy should be classified as either gestational diabetes mellitus or diabetes mellitus in pregnancy, according to WHO criteria.	Recommended
- Health-care providers should ask all pregnant women about their tobacco use (past and present) and exposure to second-hand smoke	Recommended
- Health-care providers should ask all pregnant women about their use of alcohol and other substances (past and present)	Recommended
- In high-prevalence settings, provider-initiated testing and counselling for HIV should be considered a routine component	Recommended
- In settings where the tuberculosis prevalence in the general population is 100/100 000 population or higher, systematic screening for active tuberculosis should be considered for pregnant women	Context-specific
- Daily fetal movement counting, such as with “count-to-ten” kick charts	Context-specific (research)
- Replacing abdominal palpation with symphysis-fundal height measurement for the assessment of fetal growth is not recommended to improve perinatal outcomes	Context-specific
- Routine antenatal cardiotocography is not recommended	Not Recommended
- One ultrasound scan before 24 weeks of gestation (early ultrasound) is recommended	Recommended
- Routine Doppler ultrasound examination is not recommended	Not Recommended

Appendix 1: Transthoracic Echocardiography: Handheld, Portable and Stationary Echocardiographers

The utility of transthoracic echo is limited by the technical expertise and availability of equipment to perform and interpret the scans. Handheld 2D echocardiography has an emerging role in screening for RHD, as a focused echocardiogram can be performed with reliable accuracy and reproducibility following relatively simple training, before a more detailed, confirmatory study is conducted if the results are concerning for RHD. These palm-held devices, weigh less than 300g to 400g, and use a 3.8 mHz phased array transducer. Studies of handheld echo have demonstrated 79% sensitivity and 87% specificity for all latent RHD, improving to 98% sensitivity for definite RHD [1]. Handheld echo is less expensive, allows increased convenience, and can be largely reliant on the battery as opposed to the need for reliable electricity. In areas with high prevalence of RHD, handheld echocardiography has the capacity to diagnose RHD in asymptomatic pregnant women as part of routine antenatal care.

Portable echocardiography devices have slightly larger dimensions (size similar to a laptop or a small suitcase), preserving most of the Doppler features, and other features that are available in current stationary echocardiography machines, whilst weighing less than 5Kg, which allows them to be moved inside the clinical facility, or transported for usage in the community. This contrasts with standard higher-scale stationary platforms that are heavier, weighing >50 to 70Kg, making them more difficult to mobilize, and posing difficulties and transportation/logistics issues if use outside the hospital or clinical setting is planned [1].

References:

1. **Beaton A, Lu JC, Aliku T, Dean P, Gaur L, Weinberg J,** et al. The utility of handheld echocardiography for early rheumatic heart disease diagnosis: A field study. Eur HeartJ Cardiovasc Imaging. 2015;16(5):475–82.

Appendix 2: Original Systematic Review Protocol

The detailed protocol was pre-published on PROSPERO – 2022/ CRD42022344081 (6).

Research Question and PICO

The PICO for this question was defined by the Guideline Committee as follows:

- Population: Pregnant women in areas with high prevalence of RHD; we used the data from Watkins et al. 2017 [1] to define high-prevalence areas.
- Index Test: Handheld echocardiography during routine antenatal care; any diagnostic criteria as reported by the authors in their studies.

- Comparators:

  a: Standard echocardiography (gold standard), performed using portable ultrasound or a higher-scale stationary station

  b: when this is not available, the diagnosis can be made based solely on clinical grounds

Primary Outcomes

- Carditis-ARF based on revised Jones Criteria by American Heart Association [2]
- RHD based on World Heart Federation criteria for echocardiographic diagnosis [3]
- ARF and/or RHD
  - For diagnoses based only on clinical findings, significant apical systolic and/or basal diastolic murmur(s), clinical presence of pericarditis, or unexplained congestive heart failure, are some of the findings of interest [4].

Secondary Outcomes

- Acceptability to provider and patient
- Adverse events (any), if reported
- Time to diagnosis of carditis-ARF and/or RHD
- Time to diagnosis of carditis-ARF
- Time to diagnosis of RHD

We excluded studies that did not investigate the diagnostic performance of the index tests.

Searches, Study Selection, and Data Extraction

Searches were conducted using the following sources from their inception up to 2 October 2022:

• Embase via Ovid SP (1974–present)

• MEDLINE via Ovid SP (1946–present)

Search strategies were developed by consulting the clinicians, controlled vocabularies (Medical Subject Headings=MeSH and Excerpta Medica Tree=Emtree), literature review, and test search results. Based on the recommendations from the 2nd edition of the Cochrane Handbook for Systematic Reviews of Diagnostic Test Accuracy [5], the searches were balanced between sensitivity and specificity of the search results without applying a methodological search filter.

The search strategies were peer-reviewed by another Information Specialist before the final run. The searches were run, documented, and reported by a senior information scientist and followed the globally accepted guidelines: PRISMA 2020 [6], PRISMA-S [7], PRISMA-DTA [8], PRISMA DTA for Abstracts [9], and PRESS [10].

We contacted the authors of the studies on an as-required basis to obtain the data or information. The search expression is available for consultation in Appendix 2.

The search results were imported into EndNote 20. After removal of duplicates, the remaining records were then imported into Rayyan, for double-blind screening by two reviewers. The blinding was inactivated when the screening was finished to resolve the conflicts by a third reviewer. This Search was done as part of a global search for papers on handheld echocardiography vs standard of care for screening and or diagnosis of ARF and RHD.

Data Extraction

The following data were planned for extraction from all studies and double-checked by an independent reviewer.

- Study characteristics: authors, year of publication, country, study design, sample size, study period, setting, patient selection (random/ consecutive)
- Patient characteristics: patient type, age, sex, number studied, number detected, number of ARF (to calculate the prevalence of ARF/RHD), follow-up period, targeted condition (early or latent RHD, subclinical RHD, etc.)
- Index test details: Handheld echography device used, level of experience of the sonographer, diagnostic criteria, etc.
- Reference test details: specific reference test (clinical/traditional ultrasound) as provided by the authors
- Outcomes: sensitivity and specificity directly from papers (or calculated from the true positives, false positives, true negatives, and false negatives in the 2 × 2 tables), any adverse event (deaths, complication), time to diagnosis, acceptability to provider and patient

Quality Assessment

We planned to assess the methodological quality of the included studies using the Newcastle Ottawa Scale [11]. If enough studies are included, we will use the funnel plot to assess the publication bias based on the plot's symmetry.

Rating of the certainty of the evidence using the GRADE methodology for diagnostic tests [5, 12, 13] was planned, with GRADEpro for creating the table for the diagnostic question.

Data Synthesis Including Meta-Analyses

An overview of the available studies and demographics of women were summarised. For the meta-analysis, we summarised diagnostic accuracy statistics (sensitivity and specificity) with 95% confidence intervals in the forest plot by using a bivariate random-effects model. If possible, we fitted a summary ROC curve as described by the Cochrane Collaboration [5].

Heterogeneity was assessed via visual inspection of the forest plot. Where possible, the potential sources of heterogeneity (publication year, country) were explored using meta-regression or sensitivity analysis. Where possible, we examined the impact of covariates (i.e., level of experience, time since ARF, etc.) by including covariates in the random-effects model or using subgroup analysis. Data analysis was performed using the {meta} package for R.

Subgroup Analysis

Depending on the availability of the data, we considered conducting the following subgroup analyses:

- Subgroup by disease stage:
    - Early/ subclinical RHD vs. latent RHD
    - Symptomatic or asymptomatic RHD
    - Latent vs. clinical ARF
- Subgroup by reference tests used: clinical assessment, traditional echocardiography
- Subgroup by experience level: expert vs non-expert

References

1. **Watkins DA, Johnson CO, Colquhoun SM, Karthikeyan G, Beaton A, Bukhman G,** et al. Global, regional, and national burden of rheumatic heart disease, 1990–2015. N Engl J Med. 2017;377(8):713–722.
2. **Gewitz MH, Baltimore RS, Tani LY, Sable CA, Shulman ST, Carapetis J,** et al. Revision of the Jones Criteria for the diagnosis of acute rheumatic fever in the era of Doppler echocardiography: A scientific statement from the American Heart Association. Circulation. 2015;131(20):1806–18.
3. **Reményi B, Wilson N, Steer A, Ferreira B, Kado J, Kumar K,** et al. World Heart Federation criteria for echocardiographic diagnosis of rheumatic heart disease—an evidence-based guideline. Nat Rev Cardiol. 2012;9(5):297–309.
4. **Narula J, Chandrasekhar Y, Rahimtoola S.** Diagnosis of active rheumatic carditis. The echoes of change. Circulation. 1999;100(14):1576–81.
5. **Deeks J, Bossuyt P, Takwoingi Y, Leeflang M, Flemyng E, Mellor L.** Cochrane handbook for systematic reviews of diagnostic test accuracy. Version 2. Cochrane; 2022. Available from: https://training.cochrane.org/handbook-diagnostic-test-accuracy
6. **Page MJ, McKenzie JE, Bossuyt PM, Boutron I, Hoffmann TC, Mulrow CD,** et al. The PRISMA 2020 statement: An updated guideline for reporting systematic reviews. BMJ. 2021;372:n71.
7. **Rethlefsen ML, Kirtley S, Waffenschmidt S, Ayala AP, Moher D, Page MJ,** et al. PRISMA-S: Sn extension to the PRISMA Statement for Reporting Literature Searches in Systematic Reviews. Syst Rev. 2021;10(1):39.
8. **McInnes MDF, Moher D, Thombs BD, McGrath TA, Bossuyt PM; and the PRISMA-DTA Group;** et al. Preferred reporting items for a systematic review and meta-analysis of diagnostic test accuracy studies: The PRISMA-DTA statement. JAMA. 2018;319(4):388–396.
9. **Cohen JF, Deeks JJ, Hooft L, Salameh JP, Korevaar DA, Gatsonis C,** et al. Preferred reporting items for journal and conference abstracts of systematic reviews and meta-analyses of diagnostic test accuracy studies (PRISMA-DTA for Abstracts): Checklist, explanation, and elaboration. BMJ. 2021;372:n265.
10. **McGowan J, Sampson M, Salzwedel DM, Cogo E, Foerster V, Lefebvre C.** PRESS peer review of electronic search strategies: 2015 guideline statement. J Clin Epidemiol. 2016;75:40–6.
11. **GA Wells, B Shea, D O'Connell, J Peterson, V Welch, M Losos,** et al. The Newcastle-Ottawa Scale (NOS) for assessing the quality of nonrandomised studies in meta-analyses. The Ottawa Hospital Research Institute 2021. Available from: https://www.ohri.ca/programs/clinical_epidemiology/oxford.asp
12. **Schünemann HJ, Oxman AD, Brozek J, Glasziou P, Jaeschke R, Vist GE,** et al. Grading quality of evidence and strength of recommendations for diagnostic tests and strategies. BMJ. 2008;336(7653):1106–1110.
13. **Singh S, Chang SM, Matchar DB, Bass EB.** Grading a body of evidence on diagnostic tests. Journal of General Internal Medicine. 2012;27(1):47–55.

Appendix 3: Search Strategies

Database: Embase <1974 to 2022 September 30>

1 Rheumatic Fever/ or (Rheumatic Fever* or Rheumatoid Fever*).mp. (9699)

2 exp *Echocardiography/ or exp *Doppler Echocardiography/ or *Color Doppler Echocardiography/ or *Pulsed Doppler Echocardiography/ or exp *Speckle Tracking Echocardiography/ or exp *Stress Echocardiography/ or *Contrast Echocardiography/ or *Four Dimensional Echocardiography/ or *Intracardiac Echocardiography/ or *M Mode Echocardiography/ or *Three Dimensional Echocardiography/ or *Tissue Doppler Imaging/ or *Transesophageal Echocardiography/ or *Transthoracic Echocardiography/ or *Two Dimensional Echocardiography/ or *Three Dimensional Speckle Tracking Echocardiography/ or *Two Dimensional Speckle Tracking Echocardiography/ or *Dobutamine Stress Echocardiography/ or *Exercise Stress Echocardiography/ or (Echocardiogra* or Doppler or Cardiac Echogra* or Cardiac Scan* or Cardial Echogra* or Cardioechogra* or Echo Cardiogra* or Heart Echo Sounding or Heart Echograph* or Heart Scan* or Myocardium Scan* or Ultrasound Cardiogra* or Intra-Cardiac Ultrasound or Intracardiac Echo or Intracardiac Ultrasound or Echo Stress Test or Stress Echo Test or Stress MCE).mp. (612889)

3 1 and 2 (1633)

4 (rat or rats or mouse or mice or swine or porcine or murine or sheep or lambs or pigs or piglets or rabbit or rabbits or cat or cats or dog or dogs or cattle or bovine or monkey or monkeys or trout or marmoset$1).ti. and animal experiment/ (1167889)

5 Animal experiment/ not (human experiment/ or human/) (2451550)

6 4 or 5 (2513280)

7 3 not 6 (1627)

Database: Ovid MEDLINE(R) ALL <1946 to September 30, 2022>

1 Rheumatic Heart Disease/ or exp Rheumatic Fever/ or (Rheumatic Card* or Rheumatic Fever* or Rheumatic Heart or Rheumatoid Fever* or Rheumatic Valv* or Rheumatic Pancarditis or Rheumatic Endocarditis or Rheumatic Myocarditis or Rheumatic Pericarditis or Rheumatoid Pancarditis or Rheumatoid Endocarditis or Rheumatoid Myocarditis or Rheumatoid Pericarditis or Rheumatoid Card* or Rheumatoid Heart or Rheumatoid Valv*).mp. (26046)

2 exp Echocardiography/ or exp Echocardiography, Doppler/ or Echocardiography, Three-Dimensional/ or Echocardiography, Doppler, Color/ or Echocardiography, Doppler, Pulsedor/ or Echocardiography, Stress/ or Echocardiography, Four-Dimensional/ or Echocardiography, Transesophageal/ or (Echocardiogra* or Doppler or Cardiac Echogra* or Cardiac Scan* or Cardial Echogra* or Cardioechogra* or Echo Cardiogra* or Heart Echo Sounding or Heart Echograph* or Heart Scan* or Myocardium Scan* or Ultrasound Cardiogra* or Intra-Cardiac Ultrasound or Intracardiac Echo or Intracardiac Ultrasound or Echo Stress Test or Stress Echo Test or Stress MCE).mp. (326513)

3 1 and 2 (2769)

4 exp Animals/ not Humans.sh. (5052290)

5 3 not 4 (2766)

Conference Proceedings Citation Index-Science (CPCI-S; 1990 - present)

(Rheumatic Card* or Rheumatic Fever* or Rheumatic Heart or Rheumatoid Fever* or Rheumatic Valv* or Rheumatic Pancarditis or Rheumatic Endocarditis or Rheumatic Myocarditis or Rheumatic Pericarditis or Rheumatoid Pancarditis or Rheumatoid Endocarditis or Rheumatoid Myocarditis or Rheumatoid Pericarditis or Rheumatoid Card* or Rheumatoid Heart or Rheumatoid Valv*) AND (Echocardiogra* or Doppler or Cardiac Echogra* or Cardiac Scan* or Cardial Echogra* or Cardioechogra* or Echo Cardiogra* or Heart Echo Sounding or Heart Echograph* or Heart Scan* or Myocardium Scan* or Ultrasound Cardiogra* or Intra-Cardiac Ultrasound or Intracardiac Echo or Intracardiac Ultrasound or Echo Stress Test or Stress Echo Test or Stress MCE) (Topic) 172
